# On Estimation of Genome Composition in Genetically Admixed Individuals Using Constrained Genomic Regression

**DOI:** 10.3389/fgene.2018.00185

**Published:** 2018-05-29

**Authors:** Vinzent Boerner, Dörte Wittenburg

**Affiliations:** ^1^Animal Genetics and Breeding Unit, University of New England, Armidale, NSW, Australia; ^2^Institute of Genetics and Biometry, Leibniz Institute for Farm Animal Biology, Dummerstorf, Germany

**Keywords:** genome, population, stratification, admixture, estimation, regression, constrained

## Abstract

Quantifying the population stratification in genotype samples has become a standard procedure for data manipulation before conducting genome wide association studies, as well as for tracing patterns of migration in humans and animals, and for inference about extinct founder populations. The most widely used approach capable of providing biologically interpretable results is a likelihood formulation which allows for estimation of founder genome proportions and founder allele frequency conditional on the observed genotypes. However, if founder allele frequencies are known and samples are dominated by admixed genotypes this approach may lead to biased inference. In addition, processing time increases drastically with the number of genetic markers. This article describes a simplified approach for obtaining biologically meaningful measures of population stratification at the genotype level conditional on known founder allele frequencies. It was tested on cattle and human data sets with 4,022 and 150,000 genetic markers, respectively, and proved to be very accurate in situations where founder poplations were correctly specified, or under-, over-, and miss-specified. Moreover, processing time was only marginally affected by an increase in the number of markers.

## Introduction

The quantification of population stratification in samples of genotypes is of relevance because if not accounted for it may obscure results from genome wide association studies (GWAS) (Marchini et al., 2004; Price et al., 2010). Further, it allows reconstruction of patterns of ancient migration, population segregation, and inheritance (Rasmussen et al., 2010; Kijas et al., 2012; Patterson et al., 2012; Reich et al., 2012; Skoglund et al., 2012; Hellenthal et al., 2014).

Two major approaches are used. The first approach, described by Alexander et al. (2009) as “model-based ancestry estimation,” estimates genome proportions of sampled genotypes conditional on a predefined number of ancestral populations, and is embedded in software like STRUCTURE (Pritchard et al., 2000; Falush et al., 2003; Raj et al., 2014), FRAPPE (Tang et al., 2005), and ADMIXTURE (Alexander et al., 2009). This approach yields biologically meaningful results at the individual and population levels. However, results from this algorithm still need to be appropriately incorporated into a followup GWAS (Corneveaux et al., 2010; Huson et al., 2014; Hwang et al., 2014). The second approach, classified in Alexander et al. (2009) as “algorithmic ancestry estimation,” assumes that the major structural variation among marker genotypes in samples from different populations is caused by differences in allele frequencies between populations, which can be detected and visualized by a singular value decomposition of the matrix of genetic markers. This approach, embedded in the software EIGENSTRAT (Price et al., 2006) performs GWAS within the Eigen-space on residuals formed by regressing the phenotype and the marker genotype on a predetermined number of principal components, thus removing any bias due to population stratification. However, it is not obvious how to interpret principal components in terms of ancestral population allele frequencies and individual genome proportions.

Since in many cases the population allele frequencies can be estimated from individuals of known genetic origin, Alexander and Lange (2011) provided a “supervised” mode for their method to facilitate faster genome proportion estimation for admixed individuals. However, the likelihood formulation underlying ADMIXTURE has three major disadvantages: (1) it assumes linkage equilibrium (LE) between markers, (2) it makes rather strong assumptions about the underlying distribution of genetic markers, and (3) the processing time becomes an obstacle if the number of markers is very large.

This article describes a non-linear optimisation method, called constrained genomic regression (CGR), for the estimation of founder genome proportions in marker genotypes of admixed individuals which overcomes the speed limitation and distributional assumption of the likelihood based method and proved to be robust against the violation of the LE assumption. Similar to the likelihood based method, CGR results have a biologically meaningful interpretation even if the number of possible founder populations is very large. The algorithm was tested on two data sets, a cattle data set and a human data set, and results were compared to ADMIXTURE results when the “supervised” mode was used.

## Methods

### Model

Assume that *N* populations may have contributed to an individual's genotype which can be observed at *M* bi-allelic genetic markers. Let *p*_*i,k*_ be the frequency of the minor allele of marker *i* = 1, .., *M* in population *k* = 1, .., *N*. Then, the expected count of the minor allele at marker locus *i* in individual *j* = 1, .., *L* is given as:

(1) $$E(y_{i,j})=\sum_{k=1}^{N}2p_{i,k}b_{k,j}$$

where *b*_*k, j*_ denotes the probability of contribution of population *k* to individual *j*; this parameter is typically unknown. Hence, regarding all markers jointly, the vector of allele contents *y*_*j*_ = (*y*_1,*j*_, *y*_2,*j*_, *y*_*i, j*_, .., *y*_*M,j*_) of individual *j* can be modelled using a linear regression approach:

(2) $$y_{j}=Xb_{j}+e_{j}$$

where *b*_*j*_ = (*b*_1,*j*_, .., *b*_*k,j*_, .., *b*_*N,j*_) is a vector of regression coefficients unique to individual *j*, *X* is a column matrix of dimension *M* × *N* with column *k* containing vector *X*_:,*k*_ = (2*p*_1,*k*_, .., 2*p*_*i,k*_, .., 2*p*_*M,k*_), and *e*_*j*_ = (*e*_1,*j*_, .., *e*_*i,j*_, .., *e*_*M,j*_) are the random error terms assumed to be independent and identically distributed with expectation 0. As independence between admixed individuals is assumed, model 2 is applied to each individual separately to estimate its regression coefficients.

Minimizing ${\left(y_{j}-Xb_{j}\right)}^{\prime }\left(y_{j}-Xb_{j}\right)$ would yield an ordinary least square solution for *b*_*j*_. Since the parameter space of values in *b*_*j*_ is un-constrained in model 2, regression coefficients may become negative. To ensure that the coefficients can be interpreted as proportions the parameter space of *b*_*j*_ must be constrained to the interval between 0 and 1, and the sum over *b*_*j*_ to be equal to 1. Thus, the regression coefficients have to fulfill:

(3) $${\hat{b}}_{j}=\;\underset{b_{j}}{\text{arg min}}{\left(y_{j}-Xb_{j}\right)}^{\prime }(y_{j}-Xb_{j})$$

s.t.

(4) $$b_{k,j}\geq 0\text{ for }k=1,..,N$$

(5) $$\sum_{k}^{N}b_{k,j}=1$$

Due to the constraints, particularly 4, a solution for 3 cannot be found by inverting the *X*′*X*. Instead, an iterative non-linear optimisation solver may be used.

Note that constraint 5 can be changed to:

(6) $$\sum_{k}^{N}b_{k,j}\leq 1,$$

which provides greater flexibility in situations where the number of founder populations in the model is lower than the number of founder populations which may have possibly contributed to an admixed individual, or where the allele frequency vector of a putative founder population is miss-specified (e.g., due to genotyping errors or genotype sampling).

### Data

The above algorithm was tested with two different data sets, a commercial Australian beef cattle data set, and the publicly available human data set (Gibbs et al., 2003).

#### Cattle data

The cattle data set consisted of 11,639 individuals from 11 different breed populations (breeds):“Brahman” (1,492), “Angus” (1,473), “Murray Grey” (316), “Limousin” (1,395), “Charolais” (899), “Hereford” (1,500), “Simmental” (337), “Shorthorn” (1,126), “Wagyu” (1,497), “Droughtmaster” (130), and “Santa Gertrudis” (1,474). Breeds can be grouped to European Continental *Bos Taurus* breeds (“Limousin,” “Charolais,” and “Simmental”), British *Bos Taurus* breeds (“Angus,” “Murray Grey,”, “Hereford,” and “Shorthorn”), Asian *Bos Taurus* breeds (“Wagyu”), composite breeds with predominantly *Bos Indicus* genome (“Brahman”), and composite breeds with varying proportions of *Bos Indicus* and *Bos Taurus* (“Santa Gertrudis” and “Droughtmaster”).

**Figure 1:**
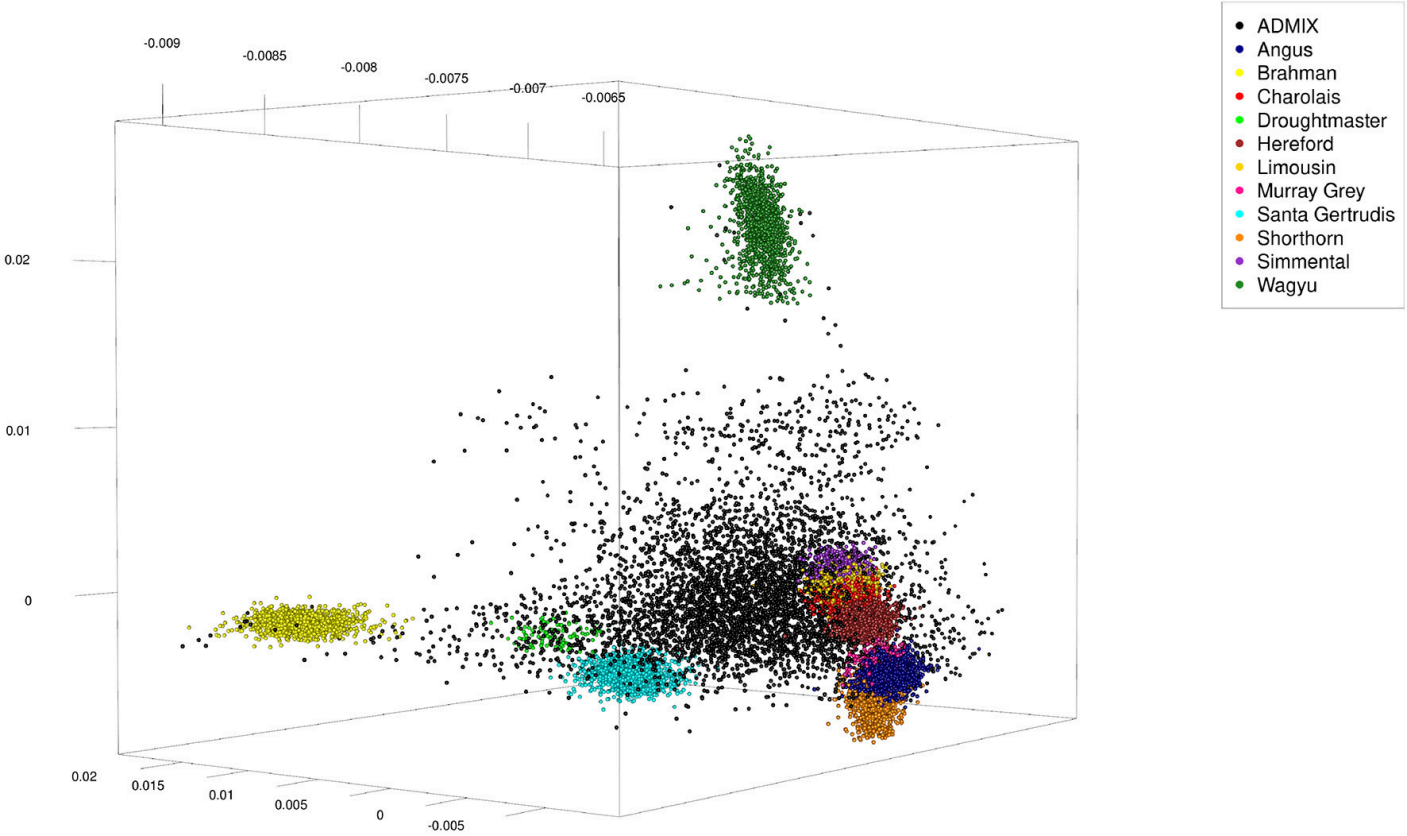
First three principal components of artificially admixed (black spheres) and non-admixed (colored spheres) animals of the cattle data set obtained from a singular value decomposition of a genetic marker matrix of dimension 16,639 (number of genotypes) by 4,022 (number of markers). The genotype sample consisted of 5,000 artificially admixed and 11,639 original genotypes.

Because genotypes of these animals were from different single nucleotide polymorphism (SNP) panels, 4,022 SNP were selected which were in common across panels. The SNP genotypes were randomly phased to obtain haplotypes, and admixed individuals were generated over five rounds. In round one the sex was randomly assigned to the 11,639 pure-bred animals and 1,000 males and 1,000 females were randomly chosen (with replacement) to serve as parents. From each pair of parents one offspring was generated from simulated gametes generated from haplotypes assuming 5 or 25 randomly located cross-overs. In the subsequent four rounds the 2,000 parents were selected among the previous 1,000 offspring, implying more than one offspring per parent. Thus, the total number of artificial admixed offspring was 5,000 (see Table 1 for a summary statistic of their genome constitution, and Figure 1 for a plot of the first three principal components obtained from a singular value decomposition of the marker matrix).

**Table 1:** Summary of number of admixed animals with genome proportions of 1 to 11 original populations.

**Number of cross-overs**	**Number of original populations contributing to an admixed genome**										
	**1**	**2**	**3**	**4**	**5**	**6**	**7**	**8**	**9**	**10**	**11**
5	121	970	465	594	424	529	584	618	477	205	13
25	120	968	453	610	394	478	465	559	576	312	65

*Note that table rows sum up to 5,000, which is the number of admixed individuals*.

#### Human data

**Figure 2:**
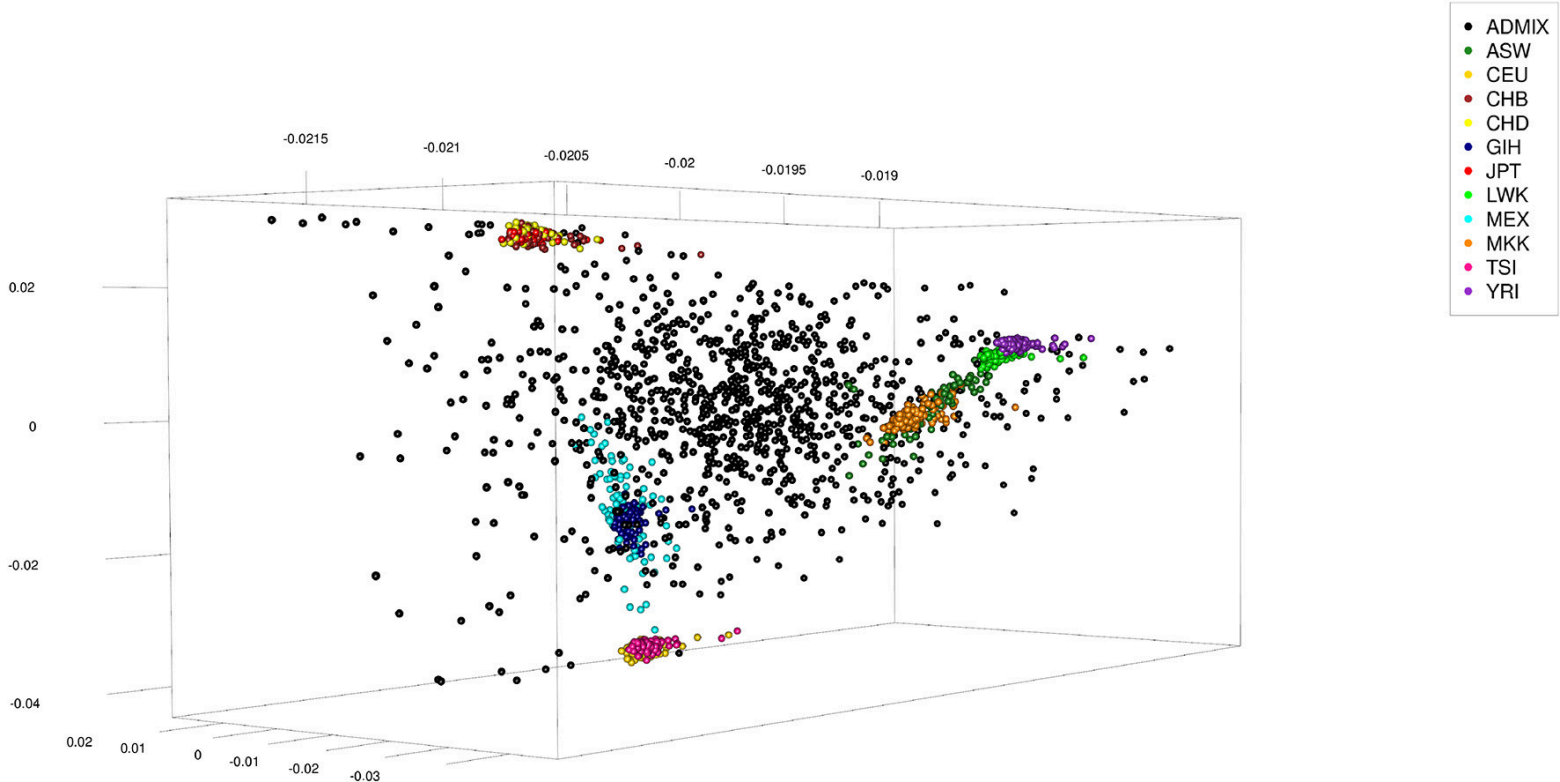
First three principal components of artificially admixed (black spheres) and non-admixed (colored spheres) individuals of the Human data set obtained from a singular value decomposition of a genetic marker matrix of dimension 2,397 (number of genotypes) by 150,000 (number of markers). The genotype sample consisted of 1,000 artificially admixed and 1,397 original genotypes. The original genotypes of non-admixed individuals were obtained from the International Hapmap Project.

The human data set was obtained from the International Hapmap Project (Gibbs et al., 2003), where the hapmap3_r3 consensus data set in plink format was used. This data set consisted of 1,397 persons from 11 different ethnicities: 87 persons of African ancestry in Southwest USA (ASW), 165 Utah residents with Northern and Western European ancestry from the CEPH collection (CEU), 137 Han Chinese in Beijing, China (CHB), 109 Chinese in Metropolitan Denver, Colorado (CHD), 101 Gujarati Indians in Houston, Texas (GIH), 113 Japanese in Tokyo, Japan (JPT), 110 Luhya in Webuye, Kenya (LWK), 86 persons of Mexican ancestry in Los Angeles, California (MEX), 184 Maasai in Kinyawa, Kenya (MKK), 102 Tuscan in Italy (TSI), and 203 Yoruban in Ibadan, Nigeria (West Africa) (YRI). The data set was re-formatted excluding all SNPs not coded as “A,” “C,” “T,” or “G.” Of the remaining SNPs only those which had an across ethnicities allele frequency ≤0.99 and ≥0.01 were kept, of which finally 150,000 were randomly selected to be used. To generate admixed individuals the sex was randomly assigned to the 1,397 genotypes and 200 males and 200 females were randomly chosen (with replacement) to serve as parents. Haplotypes of these parents were obtained by random phasing. From each pair of parents one offspring was generated by combining gametes which had been generated from their haplotypes assuming 5 or 25 randomly located cross-overs. This process was repeated till generation 5 using the offspring as parents (thus generating F1 to F5), with the number of expected progeny per parent increasing from one to two in subsequent generations. The final number of artificially generated admixed individuals was 1,000 (see Table 2 for a summary statistic of their genome constitution and Figure 2 for a plot of the first three principal components obtained from a singular value decomposition of the marker matrix).

**Table 2:** Summary of number of admixed persons with genome proportions of 1 to 11 original populations.

**Number of cross-overs**	**Number of original populations contributing to an admixed genome**										
	**1**	**2**	**3**	**4**	**5**	**6**	**7**	**8**	**9**	**10**	**11**
5	25	194	90	113	72	92	87	105	106	94	22
25	24	192	73	128	60	93	63	81	87	112	87

*Note that table rows sum up to 1,000, which is the number of admixed individuals*.

### Performance trials

#### Fully specified founder populations

Trials were conducted with the 25 and 5 cross-over human and cattle data sets where matrix *X* contained the expected allele content vectors of all 11 populations. The within population expected allele contents were calculated from individuals of known genetic background. Note that from Tables 1, 2 it can be inferred that this also included over-specified models where more populations were fitted than actually contributed to the admixed individual. These trials were run with CGR and ADMIXTURE in its “supervised”mode, where CGR was tested using either constraint 5 or constraint 6.

#### Under-specified founder populations

Two types of trials were conducted to evaluate the robustness of CGR in the situation where the number of specified founder populations was less than the number of founder populations which had actually contributed to an admixed individual.

In the first set of trials (type-1 trials) the vector of expected allele content of a single population was excluded from matrix *X*. These trials were run on the 25 cross-over cattle data set only, using the CGR algorithm with constraint 6. For ADMIXTURE run in its “supervised mode,” the number of founder populations was set to 10. Populations alternatively excluded were the Brahman breed, the Wagyu breed, and the Angus breed. As shown in Figure 1, these breeds were chosen because the first two are very distinctive whereas the last represents the cloud of European *Bos Taurus* breeds.

The second set of trials (type-2 trials) modeled a situation where a single contributing founder is known and specified (in terms of allele frequencies) but little knowledge exists about all other populations which may have contributed to that individual. The purpose was to evaluate how accurately CGR and ADMIXTURE can estimated the genome proportion of the known founder. These trials can also be regarded as miss-specification trials. For CGR the matrix *X* was reduced to two columns. The first column contained the expected allele content of a founder population which had contributed to the true genome composition of an artificially admixed individual with a proportion >0 %. The second column contained a vector of expected allele content calculated from 500 genotypes sampled randomly from all true founder genotypes but excluding those of the population already represented in the first column of *X*. As an example, if an admixed individual in the cattle data set had a true genome proportion of the Brahman breed >0%, the first column in *X* contained the vector of expected allele content of the Brahman breed. The second column contained a vector of expected allele contents calculated from 500 genotypes sampled randomly from the set of 11,639 true genotypes reduced by the genotypes of the Brahman breed. For ADMIXTURE the respective data files contained only the haplotypes of the known founder, labeled as population 1, the haplotypes of the 500 randomly sampled true individuals, labeled a population 2, and the haplotypes of those artificially admixed individuals of which genome contained a proportion of the founder breed > 0 %. These trials were conducted for artificially admixed individuals of the 25 cross-over cattle data set containing genome proportions of the Brahman, Wagyu, or Angus breed where the known founder was one of the latter breeds. For CGR constraint 6 was used. ADMIXTURE was run in its supervised mode with a population parameter set to 2.

### Result evaluation

#### Fully specified founder populations

Results were evaluated with the maximum estimation error (MAX) calculated as:

(7) $$max(|b_{k,j}-{\hat{b}}_{k,j}|),\;j=1,..,L;k=1,..,N,$$

with the bias calculated as:

(8) $$\frac{1}{LN}\sum_{j=1}^{L}\sum_{k=1}^{N}\left(b_{k,j}-{\hat{b}}_{k,j}\right),$$

the mean squared error (MSE) calculated as:

(9) $$\frac{1}{LN}\sum_{j=1}^{L}\sum_{k=1}^{N}{\left(b_{k,j}-{\hat{b}}_{k,j}\right)}^{2},$$

and the correlation between the true and estimated genome proportions calculated as:

(10) $$\frac{\sum_{j=1}^{L}\sum_{k=1}^{N}\left(b_{k,j}-\bar{b}\right)({\hat{b}}_{k,j}-\bar{\hat{b}})}{\sqrt{\sum_{j=1}^{L}\sum_{k=1}^{N}{\left(b_{k,j}-\bar{b}\right)}^{2}}\sqrt{\sum_{j=1}^{L}\sum_{k=1}^{N}{\left({\hat{b}}_{k,j}-\bar{\hat{b}}\right)}^{2}}}.$$

Further, differences between CGR results using constraint 5 or constraint 6 were evaluated by the correlation between the estimation errors.

#### Under-specified founder populations

Results from type-1 trials were evaluated by the correlation between the true and estimated genome proportion and with the mean absolute estimation error calculated as:

(11) $$\frac{1}{LN}\sum_{j=1}^{L}\sum_{k=1}^{N}|(b_{k,j}-{\hat{b}}_{k,j})|,$$

where the latter parameter was preferred to the MSE because it was more suitable for graphical result representation. Note that to calculate both parameters the differences between the true and the estimated genome proportion for the excluded population were not regarded. This allowed inference of whether the absence of one founder population had an effect on estimating the true genome portions of the remaining populations in *X*. Further, the correlation was calculated separately for six different categories of admixed individuals conditional on the true genome proportion of the excluded population these individuals inherited: 0%, >0% − ≤25%, >25% − ≤50%, >50% − ≤75%, >75% − <100%, and 100%.

Type-2 trials were evaluated as the trials for the fully specified founder populations, but the parameters were calculated only for the estimated genome proportions inherited from the known founder (the population in the first column of matrix *X*). In addition, a correlation between the estimation errors from CGR and ADMIXTURE was calculated.

### Software

CGR was implemented in a FORTRAN wrapper executable which called the NLopt library (Johnson, 2011). The optimization solver used the augmented Lagrangian algorithm (Conn et al., 1991) as global solver and the method of moving asymptotes (Svanberg, 2002) as a local solver. All computations were carried out on a desktop computer with an Intel(R) Core(TM) i7-3770 processor and 32 GB of memory. The interested reader may either download a Linux executable from the author's webpage, or implement the approach in R (R Development Core Team, 2011) where the NLopt library is available as a package.

For comparison the above data sets were analyzed with the likelihood based approach using the software ADMIXTURE (Alexander et al., 2009) in its “supervised” mode.

## Results

### Fully specified founder populations

#### Cattle data

**Figure 3:**
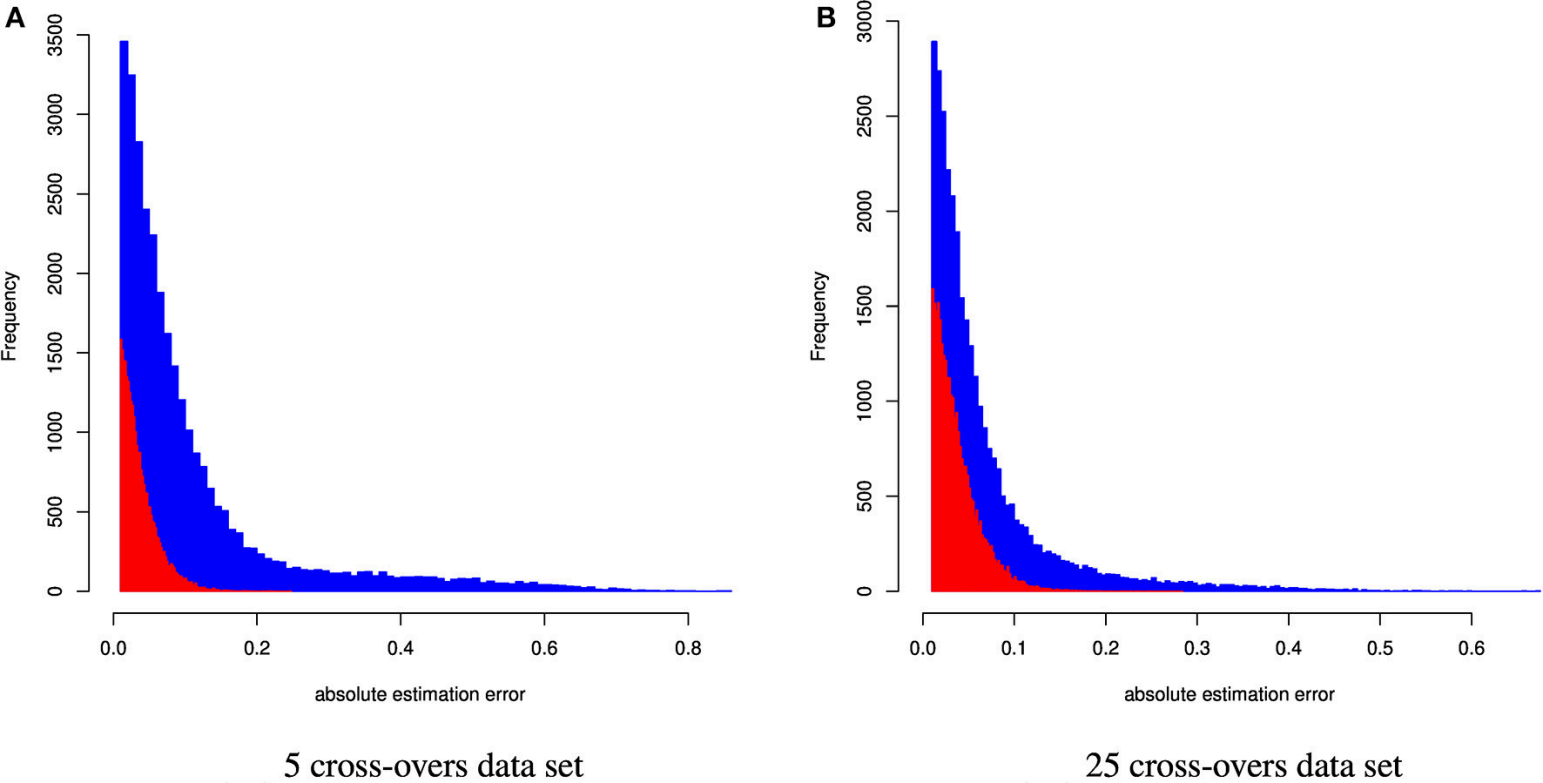
Distribution of the absolute estimation error from CGR (red) and ADMIXTURE (blue) for the cattle data set generated using 5 and 25 cross-overs and 4,022 genetic markers.

Table 3 and Figure 3 summarize the results for the admixed animals when the number of cross-overs during gametogenesis was 5 and 25, respectively. Estimates from ADMIXTURE were generally much less accurate than those from CGR. The maximum absolute estimation error produced by ADMIXTURE was 0.85 and 0.67 for the 5 and 25-crossover data set, respectively. By comparison, the same parameter for CGR reduced to 0.25 and 0.28. The same pattern was observed for the mean squared estimation error, which was for the 5-crossover data set 0.001 for CGR and 0.015 for ADMIXTURE, different by a factor of 15. While the mean squared estimation error produced by ADMIXTURE for the 25-crossover data set decreased to 1/3 of that of the 5-crossover data set, it was still five times larger than that produced by CGR. The differences in accuracy were also reflected by the correlation between the true and estimated genome proportions which were 0.63 and 0.84 for the 5 and 25-crossover data set when ADMIXTURE was used. In sharp contrast, CGR achieved a correlation of 0.97 for both data sets.

**Table 3:** Statistic of the genome proportion estimation error for the cattle data set subject to the number of cross-overs when generating admixed animals for CGR and ADMIXTURE.

**Cross-overs**	**CGR**						**ADMIXTURE**		
	**MAX_=_**	**MAX_<=_**	**MSE_=_**	**MSE_<=_**	**R_=_**	**R_<=_**	**MAX**	**MSE**	**R**
5	0.24691	0.24738	0.00103	0.00106	0.97481	0.97455	0.85393	0.01578	0.62853
25	0.28220	0.28219	0.00107	0.00106	0.97063	0.97103	0.67077	0.00566	0.84150

*MAX, maximum absolute estimation errors; MSE, mean of the squared estimation errors; R, correlation between the true and the estimated genome composition; =, CGR was run with an equality constraint; <=, CGR was run with an smaller/equality constraint. MAX and MSE were obtained across all artificially admixed individuals and all possible populations of origin*.

When constraint 5 was used, which is also used by ADMIXTURE, both methods showed no bias (results not shown). Changing to constraint 6 produced only a marginal bias of −0.00246 and −0.0014 for the 5 and 25 cross-over data set respectively. Although constraint 6 did not affect the results statistics it changed the distribution of errors as can be seen in **Figure 5**, where the effect was bigger for the 5 cross-over data set.

CGR needed 5.2 real time seconds to estimate the pure breed proportions of all 5,000 cross-bred animals, whereas ADMIXTURE needed 270 and 227 real time seconds for the 5 and 25-cross-over data set respectively, which is an increase in processing time by a factor of 50.

#### Human data

**Figure 4:**
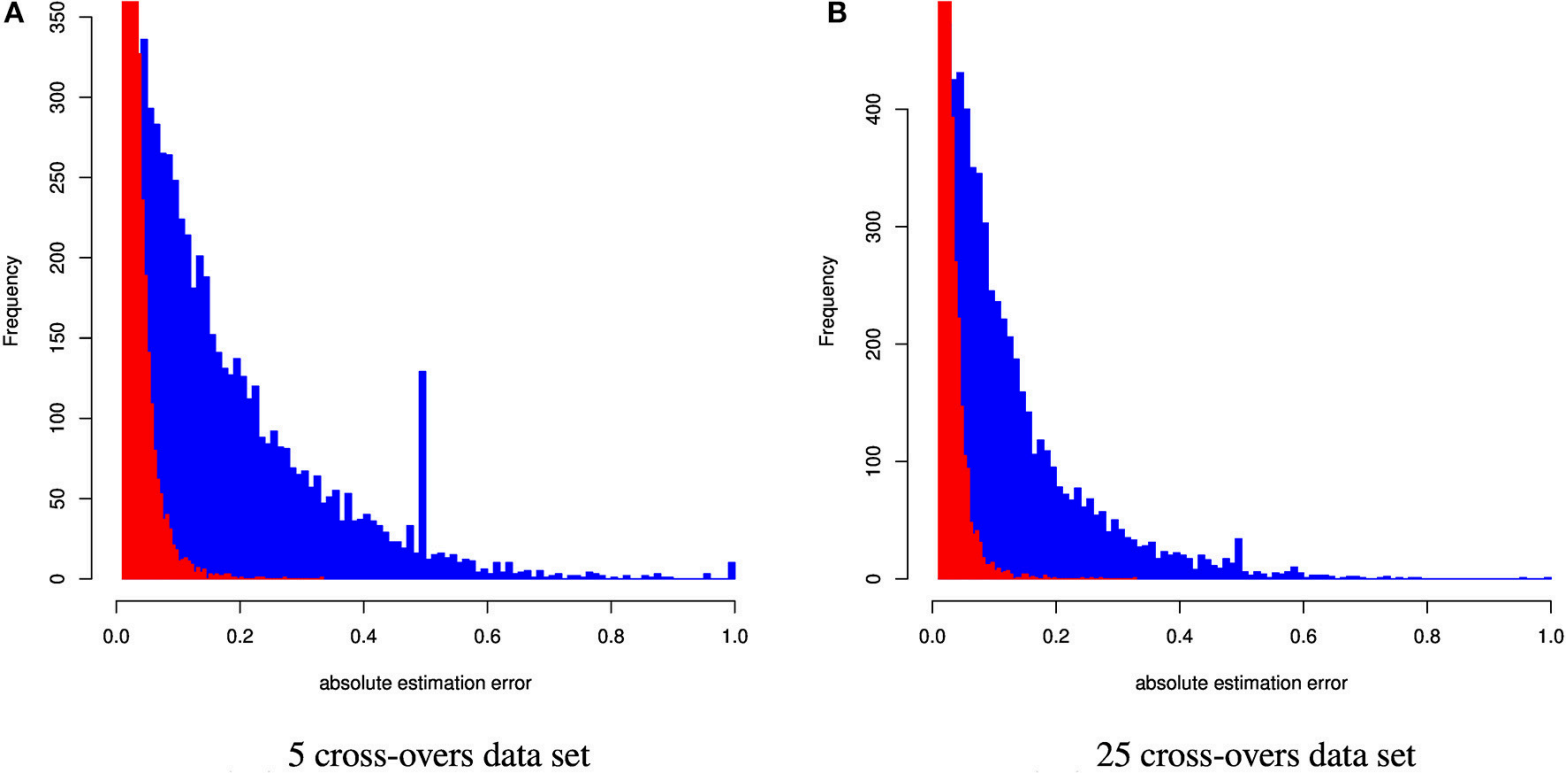
Distribution of the absolute estimation error from CGR (red) and ADMIXTURE (blue) for the human data set generated using 5 and 25 cross-overs and 150 k genetic markers.

Table 4 and Figure 4 summarize the results for the artificially admixed individuals when the number of cross-overs during gamogenesis was 5 and 25, respectively. Similar to the cattle data analysis, results from ADMIXTURE were generally much less accurate than those from CGR, but the differences increased substantially. The maximum absolute estimation error produced by ADMIXTURE was 0.99, independent of the number of cross-overs, whereas for CGR this parameter was 0.33 for both data sets. The pattern for the mean squared estimation error was similar. For ADMIXTURE the error decreased from 0.03 to 0.016 when using the 5 and 25-crossover data set respectively. However, for CGR this parameter was always much lower with 0.0007 and 0.00054 for the 5 and 25-crossover data set respectively. Thus, compared to CGR the mean squared estimation error produced by ADMIXTURE was approximately 30 times higher. As with the cattle data set, differences in accuracy were also reflected by the correlation between the true and estimated genome proportions which were 0.4 and 0.65 for the 5 and 25-crossover data set when ADMIXTURE was used, whereas CGR achieved a correlation of 0.98 independent of the data set.

**Table 4:** Statistic of the genome proportion estimation error for the human data set subject to the number of cross-overs when generating admixed individuals for CGR and ADMIXTURE.

**Cross-overs**	**CGR**						**ADMIXTURE**		
	**MAX_=_**	**MAX_<=_**	**MSE_=_**	**MSE_<=_**	**R_=_**	**R_<=_**	**MAX**	**MSE**	**R**
5	0.33489	0.33583	0.00070	0.00071	0.98245	0.98245	0.99999	0.02968	0.40357
25	0.32694	0.33060	0.00054	0.00054	0.98478	0.98468	0.99990	0.01608	0.64841

*MAX, maximum absolute estimation errors; MSE, mean of the squared estimation errors; R, the correlation between the true and the estimated genome composition; =, CGR was run with an equality constraint; <=, CGR was run with an smaller/equality constraint. MAX and MSE were obtained across all artificially admixed individuals and all possible populations of origin*.

**Figure 5:**
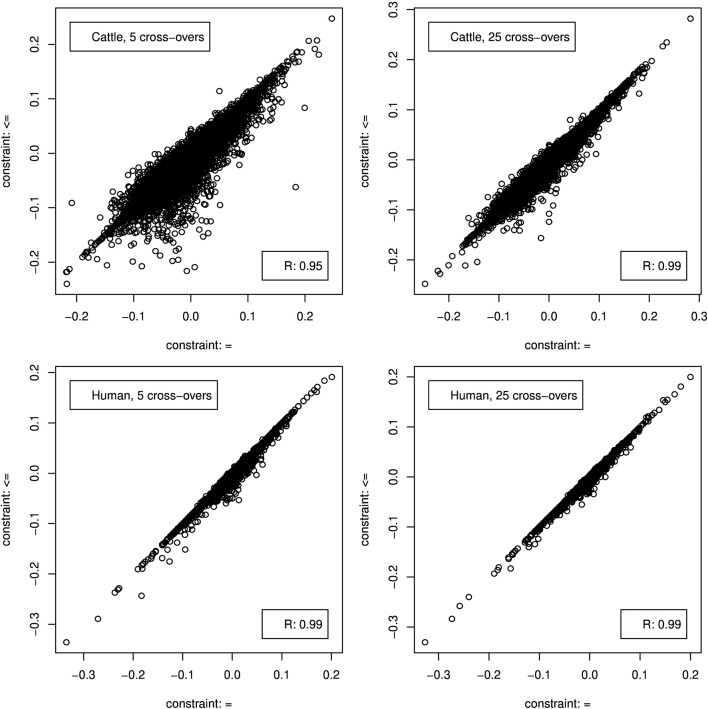
Correlation between genome composition estimation errors when CGR was used either with constraint 5 (x-axis) or 6 (y-axis).

As for the cattle data set, both methods showed not bias (results not shown) when CGR was used with constraint 5. Running CGR with constraint 6 produced only a marginal bias of −0.00124 and −0.00078 for the 5 and 25 cross-over data set respectively. By contrast to the cattle data set, constraint 6 had almost no effect on the distribution of errors (see Figure 5) for both data sets.

CGR required 26 real time seconds to estimate the founder genome proportions of all 1,000 admixed individuals. In contrast, ADMIXTURE needed 3,638 and 7,390 real time seconds for the 5 and 25-crossover data set, with the 25-crossover data set taking twice the number of iterations to converge. Thus, compared to CGR, ADMIXTURE's processing time for the 25-crossover data set was increased by factor 284.

### Under-specified founder populations

#### Type-1 trials

**Figure 6:**
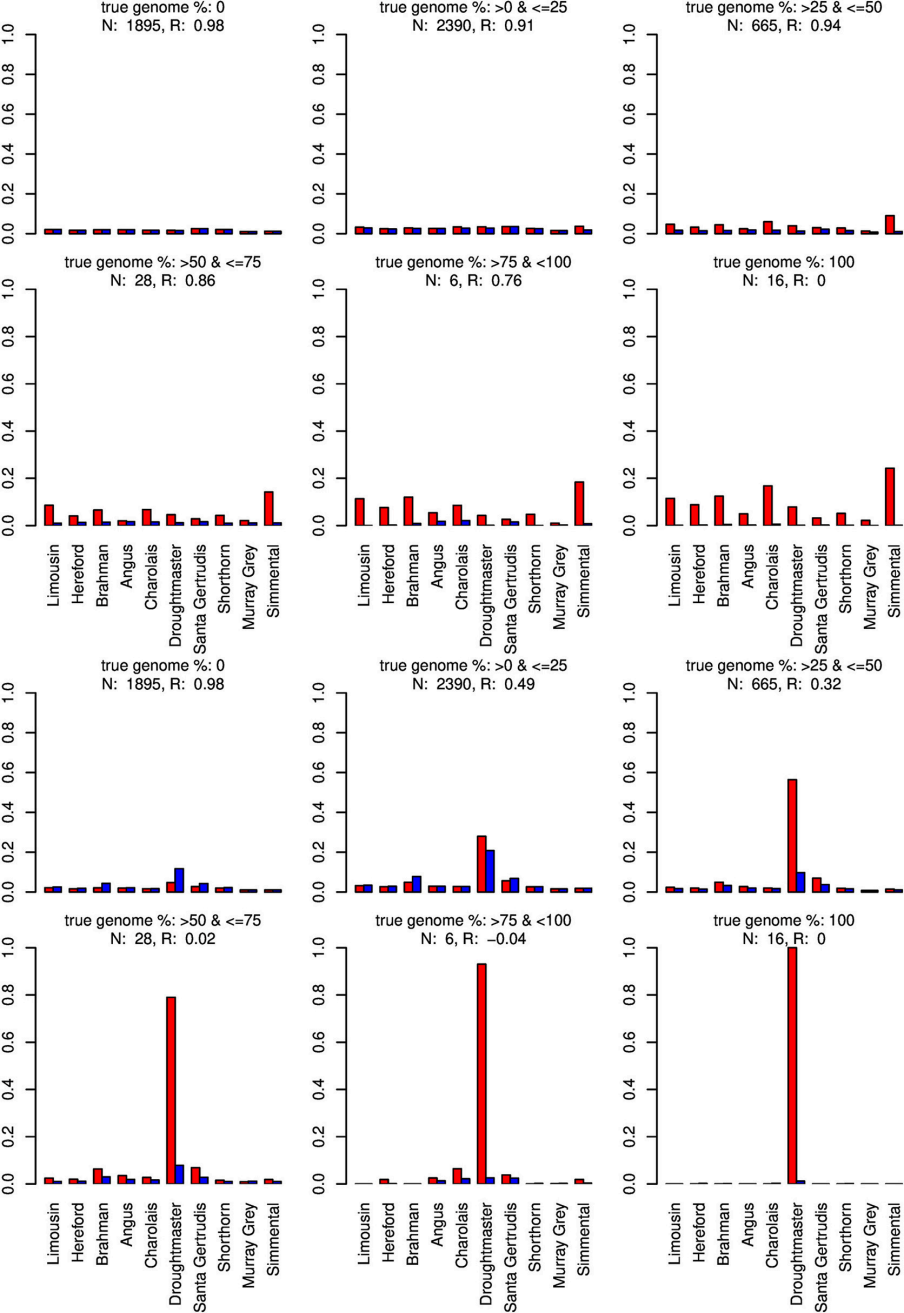
Distribution of the average absolute estimation error within possible founder breeds for the 25 cross-over cattle data set from a model using all 11 breeds (blue) and a model excluding the Wagyu breed (red). Results are shown for categories of artificially admixed animals having 0%, >0%– ≤25%, >25%– ≤50%, >50%– ≤75%, >75%– <100%, and 100% genome of the excluded breed. The graphs in the upper two rows show the results from CGR the lower two rows those from ADMIXTURE. R, correlation between the true and the estimated genome proportion when the Wagyu breed was excluded; N, number of used admixed individuals for calculating R.

**Figure 7:**
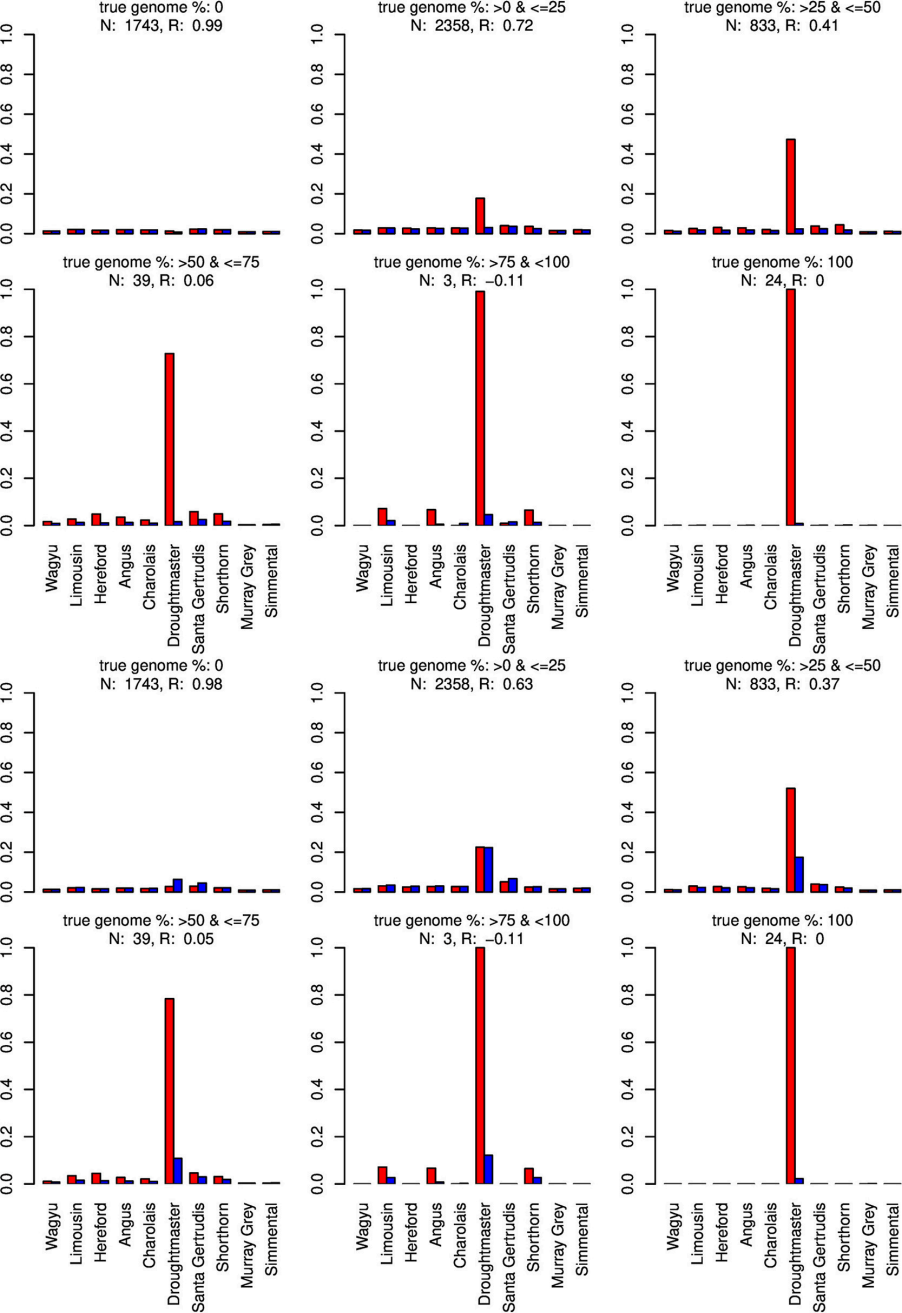
Distribution of the average absolute estimation error within possible founder breeds for the 25 cross-over cattle data set from a model using all 11 breeds (blue) and a model excluding the Brahman breed (red). Results are shown for categories of artificially admixed animals having 0%, >0%– ≤25%, >25%− ≤50%, >50%– ≤75%, >75%– <100%, and 100% genome of the excluded breed. The graphs in the upper two rows show the results from CGR the lower two rows those from ADMIXTURE. R, correlation between the true and the estimated genome proportion when the Brahman breed was excluded; N, number of used admixed individuals for calculating R.

**Figure 8:**
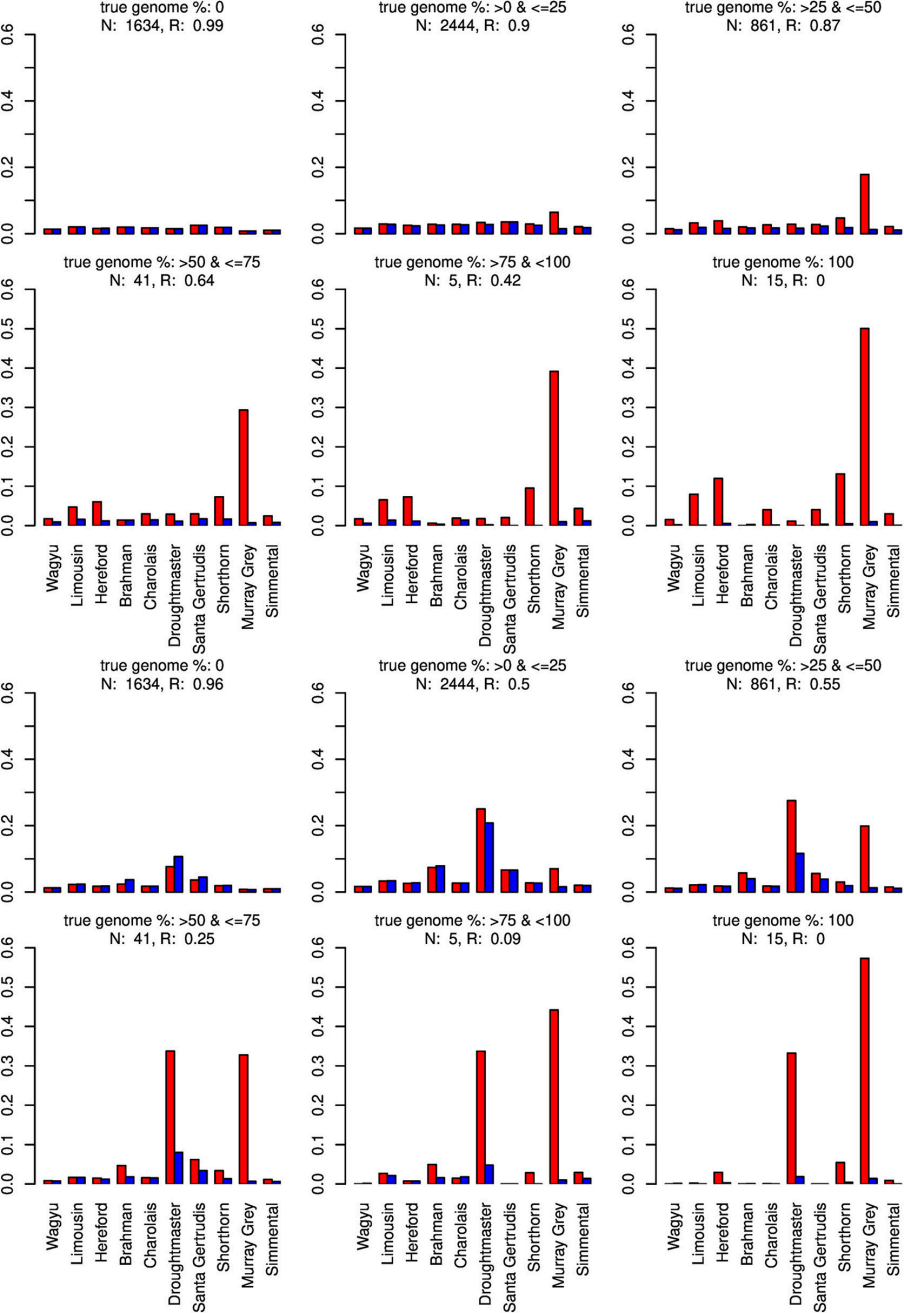
Distribution of the average absolute estimation error within possible founder breeds for the 25 cross-over cattle data set from a model using all 11 breeds (blue) and a model excluding the Angus breed (red). Results are shown for categories of artificially admixed animals having 0%, >0%– ≤25%, >25%− ≤50%, >50%– ≤75%, > 75%– <100%, and 100% genome of the excluded breed. The graphs in the upper two rows show the results from CGR the lower two rows those from ADMIXTURE. R, correlation between the true and the estimated genome proportion when the Angus breed was excluded; N, number of used admixed individuals for calculating R.

For type-1 trials a single founder population was omitted from matrix *X* and genome compositions were estimated for all artificially admixed individuals in the cattle data set. Results are summarized in Figures 6–8. As expected, admixed individuals which did not contain portions of the omitted founder population genome were not affected by its exclusion from the model when CGR was used. For ADMIXTURE the exclusion had a small deceasing effect on the mean absolute estimation error where the initial error level was higher than for CGR. For both algorithms, and invariant to the excluded breed, the correlation between the true and estimated genome proportion deteriorated with an increasing true genome proportion of the excluded population in the admixed individuals. However, result accuracy deteriorated much quicker for ADMIXTURE than for CGR, where this was most obvious when the Wagyu and Angus breed were omitted (see Figures 6, 8). For example in the category “true genome %: >25– ≤ 50” CGR achieved a correlation of 0.94 and 0.87, when the Wagyu and Angus breed was omitted, respectively. In contrast, the same results from ADMIXTURE were 0.32 and 0.55 only. The difference in the rate of deterioration was less prominent when the Brahman breed was omitted (see Figure 7).

When the Wagyu breed was omitted CGR allocated the genome proportions of this breed to the Simmental breed, which is the most related breed in terms of population allele frequency (see Figure 8). In sharp contrast, ADMIXTURE allocated the genome proportions to the Droughtmaster breed which has an allele frequency correlation to the Wagyu breed slightly lower than that of the Simmental breed.

The same pattern was observed when the Angus breed was excluded. Again CGR assigned the genome proportions to its next relative, the Murray Grey breed, whereas ADMIXTURE allocated the huge genome proportions to both, the Murray Grey breed and Droughtmaster breed.

**Figure 9:**
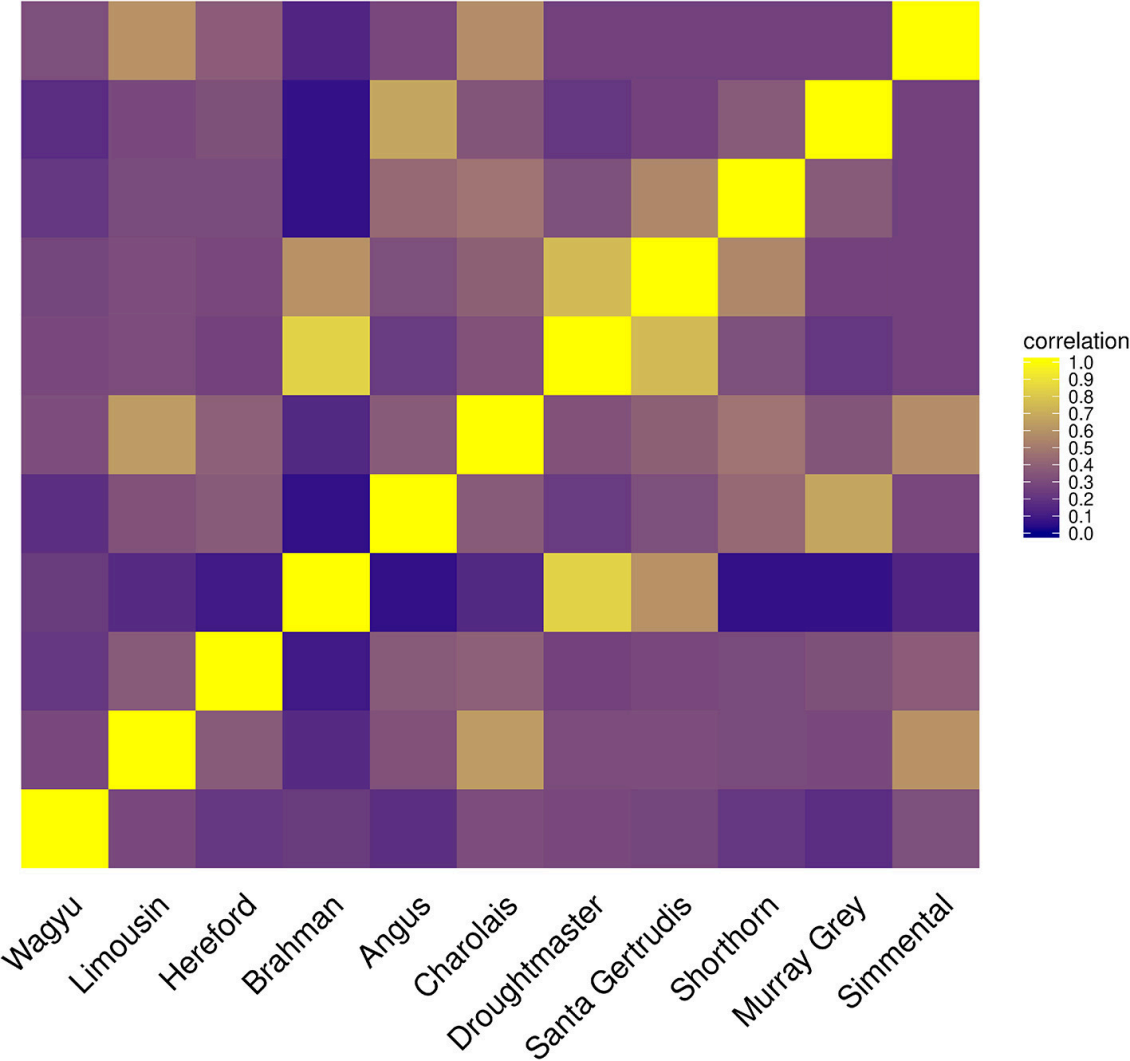
Heatmap of correlations between cattle populations calculated from their allele frequency vector.

Only when the Brahman breed was excluded, results from both algorithms showed a similar pattern assigning genome proportions to the Droughmaster breed. However, for CGR this was consistent with the observations that the Droughtmaster breed is the closest relative of the Brahman breed in terms of allele frequencies (see Figure 9).

#### Type-2 trials

Result from these trials are summarized in Table 5. For the correlation between the true and the estimated genome proportions CGR and ADMIXTURE achieved very similar results. For both algorithms the correlation was very high at 0.98, 0.95, and 0.86 for the Wagyu, Brahman, or Angus breed respectively. For CGR the mean and maximum estimation errors for the Wagyu and Brahman breed were equal to or lower than those for the fully specified model, whereas for the Angus breed these parameters were higher. For ADMIXTURE the mean squared error and maximum absolute estimation error were drastically reduced compared to fully specified model. Moreover, the very high correlation between estimation errors from CGR and ADMIXTURE shows that estimates of individual genome proportions from both algorithms were very similar.

**Table 5:** Statistic of the genome proportion estimation errors from type-2 robustness trials for the cattle data set when CGR or ADMIXTURE was used.

**Breed (N)**	**CGR**			**ADMIXTURE**			**R_err_**
	**MSE**	**MAX**	**R**	**MSE**	**MAX**	**R**	
Wagyu (3105)	0.00079	0.13434	0.98187	0.00083	0.13921	0.98300	0.95
Brahman (3257)	0.00237	0.23922	0.95150	0.00277	0.22622	0.94926	0.99
Angus (3366)	0.00664	0.41495	0.86223	0.00719	0.39024	0.85383	0.99

*For CGR the model matrix X for each artificially admixed individual which contained a proportion of genome of the founder breed in column “breed (N)” > 0 % had only 2 columns: one containing the expected allele content vector of the breed given in column “breed (N),” and one containing the expected allele content vector constructed from 500 genotypes randomly sampled from the true genotypes of the remaining 10 cattle breeds. For ADMIXTURE the respective data files contained only the haplotypes of the breed in column “breed (N),” labeled as population 1, the haplotypes of the 500 randomly sampled individuals, labelled a population 2, and the haplotypes of those artificially admixed individuals which contained a proportion of the founder breed genome in column “breed (N)” > 0 %. Thus, CGR and ADMIXTURE estimated genome proportions only for artificially admixed individuals which contained a proportion of the founder breed genome in column “breed (N)” > 0 %. breed (N): the modeled breed and the number of artificially admixed individuals which contained a proportion of the genome of that breed > 0 %. All subsequent parameters were calculated using only the estimated genome proportion of the breed given in column “breed (N).” MAX, maximum absolute estimation errors; MSE, mean of the squared estimation errors; R, the correlation between the true and the estimated genome composition; R_err_, the correlation between the estimation error from CGR and from ADMIXTURE*.

## Discussion

The modeling approach of CGR can be regarded as rather simple compared to the elaborate likelihood formulation underlying ADMIXTURE. However, results given here show that CGR outperforms ADMIXTURE in its supervised mode in terms of result accuracy and speed.

Unlike the maximum likelihood approach of Alexander et al. (2009) which searches for the parameter values making the observed allele content most likely, CGR aims at the parameter values minimizing the difference between the expected and observed allele content. The major difference between the two approaches is that the population-specific allele frequencies are assumed to be known in CGR. Thus, the dimensions of the parameter space reduce from *N*(*M* + *L*) to *NL*. This huge reduction (e.g., by 99.3 % in the human data set) leads to the computational benefit of CGR. With CGR, the number of parameters to be estimated is typically smaller than the number of observations (*NL* < *ML*), which is not necessarily the case for ADMIXTURE (*N*(*M* + *L*) > *ML*). Thus, due to the high model complexity, not all parameters can be identified from the likelihood and the error of parameter estimates is increased. Further, CGR requires less severe assumptions than ADMIXTURE, which may have also improved the precision of estimation as seen from the comparison of estimation errors. ADMIXTURE assumes that the distribution of the minor allele is binomial whereas CGR makes no assumptions about the underlying distribution. In addition, the likelihood calculation of ADMIXTURE explicitly requires marker independence (linkage equilibrium) which is relaxed in CGR because linear dependencies may occur in the row space of the design matrix *X*. To let CGR explicitly accounting for linkage disequilibrium it must be extended to a generalized least square formulation which allows for a non-diagonal co-variance matrix between residuals. Omitting this matrix affects the efficiency of the estimator but not the unbiasedness. This might be one reason for the accurate CGR results for the 5 cross-over data sets. However, both approaches assume Hardy-Weinberg equilibrium, ADMIXTURE for obtaining genotype frequencies based on the binomial distribution and CGR for deriving the expected allele content.

Another factor contributing to the inferior performance of ADMIXTURE in these examples may be due to ADMIXTURE re-estimating *p*_*i, k*_ frequencies in every single iteration using *y*_*j*_ vectors with known founder populations, as well as *y*_*j*_ vectors weighted by their respective *b*_*k, j*_, whereas CGR regards *p*_:, *k*_ vectors as constant. Omitting the iterative procedure of re-estimated allele frequencies in its supervised mode, the dimensionality of ADMIXTURE's parameter space would have been equal to that of CGR, and ADMIXTURE might have performed similar to CGR. However, with likelihoods being demanding to compute the difference in speed may have remained.

The method of constrained genomic regression has already been used for parentage verification (Boerner, 2017), where it was found that an additional variable of sufficient correlation to *y* must be fitted to account for an insufficient model fit. That is, it cannot be assumed that a poorer model fit will automatically result in decreased coefficients in *b* when CGR is run with constraint 6. A similar pattern was found for type-1 trials where the number of founder populations was under-specified. CGR almost always exploited the upper boundary of 1 for $\overset{N}{\underset{k}{\sum }}b_{k,j}$ even when the genome of admixed individual contained large proportions of the genome of the excluded population (results not shown). One possible explanation is the phylogeny of cattle populations which is also expressed by the correlation between their allele frequency vectors (see Figure 9). For example, the Simmental breed is able to explain a certain proportion of the Wagyu breed because there was a substantial importation of Simmental cattle into Japan in the early 1900s. The Drougthmaster breed is the main attractor for Brahman genome proportions if the Brahman breed is excluded because Droughtmaster was developed from various breeds where *Bos inidcus* had a major influence. This is also reflected by their very high allele frequency correlation of >0.8. The same holds for the Angus breed where the Murray Grey breed is the closest relative. Because of the between population correlation patterns, an additionally fitted variable could not prevent CGR from moving genome proportions to the closest relative, where the additionally fitted variable was a vector of ones or a vector of randomly sampled allele frequencies expressing the lack of knowledge about the missing population.

The type-1 under-specification trials were also conducted with ADMIXTURE, were the accuracy of the ADMIXTURE results was inferior to the accuracy of CGR results. While this was consistent with the pattern observed in trials with fully specified founder populations, the ADMIXTURE genome allocation in type-1 trials deserves attention. Contrarily to CGR, ADMIXTURE assigned huge genome proportions to the Droughtmaster breed almost invariant of whether the Wagyu, Brahman, or Angus breed was excluded from *X*. Figures 6–8 also reveal that this was the case for fully specified trials as well. As pointed out above, ADMIXTURE re-estimates allele frequencies in every single iteration. In combination with the fact that the Droughtmaster breed had the smallest number of individuals in the sample and is located in the center of the singular value plot (see Figure 1), its allele frequencies derived from the true genotypes have very little weight when allele frequencies are re-estimated using founder and admixed individuals. Thus, re-estimating allele frequencies in an attempt to minimize a goal function is not always desirable, and for the trials presented here might be the reason for the inferior performance of ADMIXTURE.

Results from type-2 robustness trials reveal that a mean allele content vector calculated from 500 genotypes sampled from all possible contributing founder populations but excluding the focused founder population was sufficient to estimate the genome proportions inherited from focused founder populations with high accuracy. This holds for both CGR and ADMIXTURE. However, both algorithms failed when the mean vector was replaced by a vector of ones or a vector of randomly drawn allele contents, where using CGR with constraint 6 had no effect (results not shown). An interesting aspect of these results is that the maximum absolute estimation error and mean squared estimation error generated by ADMIXTURE were drastically reduced compared to the fully specified model which might be a result of the reduced parameter space in type-2 trials. The results from type-2 trials are of particular relevance for settings where a researcher or an animal or plant breeding organization is only interested in the genome proportion of a particular founder population which is precisely specified in terms of allele frequencies. If a mean allele frequency of all other possible contributing founder populations or their close phylogenetic relatives can be specified, the thought after genome proportion can be estimated with high precision.

CGR can be efficiently applied to dense marker sets but a sufficiently large training set consisting of individuals with known origin is required to estimate the allele frequencies in advance.

In addition to the superior speed and accuracy of CGR, results given here support that the algorithm is robust against over-, under-, and miss-specification of founder populations. Thus genome proportions of present or correctly specified founder populations are estimated with high precision when the missing population is not dominating the genome. Moreover, CGR also provides highly accurate results when only one founder population is available and a mean allele frequency is specified for all other potentially contributing populations.

## Author contributions

VB developed the algorithm, wrote the computer program, carried out the simulation, and wrote and revised manuscript. DW contributed to the theoretical investigations and revised the manuscript.

### Conflict of interest statement

The authors declare that the research was conducted in the absence of any commercial or financial relationships that could be construed as a potential conflict of interest.

## References

[B1] Alexander D. H. Lange K. (2011). Enhancements to the admixture algorithm for individual ancestry estimation. BMC Bioinformatics 12:246. 10.1186/1471-2105-12-246 21682921 PMC3146885

[B2] Alexander D. H. Novembre J. Lange K. (2009). Fast model-based estimation of ancestry in unrelated individuals. Genome Res. 19, 1655–1664. 10.1101/gr.094052.109 19648217 PMC2752134

[B3] Boerner V. (2017). On marker-based parentage verification via non-linear optimization. Genet. Sel. Evol. 49:50. 10.1186/s12711-017-0324-3 28619083 PMC5472000

[B4] Conn A. R. Gould N. I. Toint P. (1991). A globally convergent augmented lagrangian algorithm for optimization with general constraints and simple bounds. SIAM J. Numer. Anal. 28, 545–572.

[B5] Corneveaux J. J. Myers A. J. Allen A. N. Pruzin J. J. Ramirez M. Engel A.. (2010). Association of cr1, clu, and picalm with alzheimer's disease in a cohort of clinically characterized and neuropathologically verified individuals. Hum. Mol. Genet. 19, 3295–3301. 10.1093/hmg/ddq221 20534741 PMC2908469

[B6] Falush D. Stephens M. Pritchard J. K. (2003). Inference of population structure using multilocus genotype data: linked loci and correlated allele frequencies. Genetics 164, 1567–1587. 12930761 10.1093/genetics/164.4.1567 PMC1462648

[B7] Gibbs R. A. Belmont J. W. Hardenbol P. Willis T. D. Yu F. Yang H. (2003). The international hapmap project. Nature 426, 789–796. 10.1038/nature02168 14685227

[B8] Hellenthal G. Busby G. B. Band G. Wilson J. F. Capelli C. Falush D.. (2014). A genetic atlas of human admixture history. Science 343, 747–751. 10.1126/science.1243518 24531965 PMC4209567

[B9] Huson H. J. Kim E.-S. Godfrey R. W. Olson T. A. McClure M. C. Chase C. C.. (2014). Genome-wide association study and ancestral origins of the slick-hair coat in tropically adapted cattle. Front. Genet. 5:101. 10.3389/fgene.2014.00101 24808908 PMC4010767

[B10] Hwang E.-Y. Song Q. Jia G. Specht J. E. Hyten D. L. Costa J.. (2014). A genome-wide association study of seed protein and oil content in soybean. BMC Genomics 15:1. 10.1186/1471-2164-15-1 24382143 PMC3890527

[B11] Johnson S. G. (2011). The nlopt Nonlinear-Optimization Package.

[B12] Kijas J. W. Lenstra J. A. Hayes B. Boitard S. Porto Neto L. R. San Cristobal M.. (2012). Genome-wide analysis of the world's sheep breeds reveals high levels of historic mixture and strong recent selection. PLoS Biol. 10:e1001258. 10.1371/journal.pbio.1001258 22346734 PMC3274507

[B13] Marchini J. Cardon L. R. Phillips M. S. Donnelly P. (2004). The effects of human population structure on large genetic association studies. Nat. Genet. 36, 512–517. 10.1038/ng1337 15052271

[B14] Patterson N. Moorjani P. Luo Y. Mallick S. Rohland N. Zhan Y.. (2012). Ancient admixture in human history. Genetics 192, 1065–1093. 10.1534/genetics.112.145037 22960212 PMC3522152

[B15] Price A. L. Patterson N. J. Plenge R. M. Weinblatt M. E. Shadick N. A. Reich D. (2006). Principal components analysis corrects for stratification in genome-wide association studies. Nat. Genet. 38, 904–909. 10.1038/ng1847 16862161

[B16] Price A. L. Zaitlen N. A. Reich D. Patterson N. (2010). New approaches to population stratification in genome-wide association studies. Nat. Rev. Genet. 11, 459–463. 10.1038/nrg2813 20548291 PMC2975875

[B17] Pritchard J. K. Stephens M. Donnelly P. (2000). Inference of population structure using multilocus genotype data. Genetics 155, 945–959. 10835412 10.1093/genetics/155.2.945 PMC1461096

[B18] R Development Core Team (2011). R: A Language and Environment for Statistical Computing. Vienna: R Foundation for Statistical Computing.

[B19] Raj A. Stephens M. Pritchard J. K. (2014). faststructure: variational inference of population structure in large snp data sets. Genetics 197, 573–589. 10.1534/genetics.114.164350 24700103 PMC4063916

[B20] Rasmussen M. Li Y. Lindgreen S. Pedersen J. S. Albrechtsen A. Moltke I.. (2010). Ancient human genome sequence of an extinct palaeo-eskimo. Nature 463, 757–762. 10.1038/nature08835 20148029 PMC3951495

[B21] Reich D. Patterson N. Campbell D. Tandon A. Mazieres S. Ray N.. (2012). Reconstructing native american population history. Nature 488, 370–374. 10.1038/nature11258 22801491 PMC3615710

[B22] Skoglund P. Malmström H. Raghavan M. Storå J. Hall P. Willerslev E.. (2012). Origins and genetic legacy of neolithic farmers and hunter-gatherers in europe. Science 336, 466–469. 10.1126/science.1216304 22539720

[B23] Svanberg K. (2002). A class of globally convergent optimization methods based on conservative convex separable approximations. SIAM J. Optim. 12, 555–573. 10.1137/S1052623499362822

[B24] Tang H. Peng J. Wang P. Risch N. J. (2005). Estimation of individual admixture: analytical and study design considerations. Genet. Epidemiol. 28, 289–301. 10.1002/gepi.20064 15712363

